# Point‐of‐Care Lung Ultrasound in Ambulatory Heart Failure—Moving From Feasibility to Outcomes

**DOI:** 10.1002/clc.70342

**Published:** 2026-05-06

**Authors:** Shaher Yar, Khadija Sana, Aiman Aijaz, Parvat Kuwar Chhetri

**Affiliations:** ^1^ University College of Medicine and Dentistry Lahore Pakistan; ^2^ T.M.S.S Medical college Bogura Bangladesh

**Keywords:** ambulatory care, automated B‐line counter, B‐lines, heart failure, lung ultrasound, point‐of‐care ultrasound, pulmonary congestion

To the Editor,

Golombeck et al. deserve credit for examining point‐of‐care lung ultrasound (LUS) in the ambulatory heart failure (HF) setting and showing that a structured training pathway can yield diagnostic‐quality scans within routine clinic workflows [1]. In their cohort of 75 patients, novice HF clinicians achieved good image quality in 88% of studies, and LUS detected mild pulmonary congestion that clinical volume assessment alone did not reliably capture. This work strengthens the argument for integrating LUS into outpatient HF care, where sub‐clinical congestion is common and physical examination carries well‐documented limitations.

The reproducibility findings are the study's most consequential observation. Even among experts, B‐line quantification agreement was only moderate, and concordance between manual expert counts and the automated B‐line counter (ABLC) in the Butterfly IQ+ probe was similarly modest. B‐lines are established surrogates for extravascular lung water and carry prognostic weight across acute and chronic HF [2, 3]. Platz et al. found that residual B‐lines at discharge were associated with a fivefold increase in readmission or death in acute HF, while even modest B‐line elevations identified ambulatory patients at nearly fourfold risk of hospitalization at 6 months [2]. These data support the authors' implication that a standardized automated approach could reduce inter‐observer variability and enable practical longitudinal congestion tracking across clinicians and sites.

The outpatient context matters. The CLUSTER‐HF trial, a single‐blinded randomized controlled trial of 126 patients, found that providing LUS results to treating clinicians reduced the composite of urgent HF visits, re‐hospitalization, and death by 45% over 6 months, driven primarily by fewer urgent visits [4]. Separately, Marini et al. showed that LUS‐guided diuretic adjustment in 244 patients with chronic HF reduced acute decompensation hospitalizations by 56% at 90 days, with accompanying improvements in NT‐proBNP and quality‐of‐life scores [5]. The current feasibility study raises the logical next question: can ABLC‐guided management replicate or improve these outcomes while lowering training demands and improving scalability?

Several methodological considerations should shape future work. B‐lines are not HF‐specific; interstitial lung disease and pneumonia both elevate counts, arguing for protocols that integrate clinical context and, where needed, focused cardiac or venous ultrasound. The underpowered exploratory comparisons with NT‐proBNP, weight, and pulmonary artery diastolic pressure point toward a “multi‐sensor” strategy in which LUS complements rather than replaces implantable hemodynamic monitoring. The CHAMPION trial established that CardioMEMS‐guided care reduces HF hospitalizations versus clinical assessment alone, reinforcing the rationale for combining tools that capture congestion through different mechanisms [6].

Golombeck et al. have demonstrated that outpatient LUS is feasible for novice providers and that automated quantification may reduce variability and improve subclinical congestion detection. What the field now needs is a well‐powered randomized trial comparing ABLC‐guided care against standard management and expert‐read LUS, with patient‐centered endpoints including urgent visits, HF hospitalizations, and diuretic‐related adverse events.

## Author Contributions

**Shaher Yar:** conceptualization, writing − original draft, writing – review and editing. **Khadija Sana:** literature review, writing – original draft. **Aiman Aijaz:** Literature review, writing – review and editing. **Parvat Kuwar Chhetri:** writing – original draft, writing – review and editing.

## Funding

The authors have nothing to report.

## Conflicts of Interest

The authors declare no conflicts of interest.

## Data Availability

The authors have nothing to report.
